# Clinical effectiveness of an ultra-brief intervention for common mental health syndromes in primary care: study protocol for a cluster randomized controlled trial

**DOI:** 10.1186/s13063-015-0778-y

**Published:** 2015-06-05

**Authors:** Sunny Collings, Fiona Mathieson, Anthony Dowell, James Stanley, Simon Hatcher, Felicity Goodyear-Smith, Brigitte Lane, Amy Munsterman

**Affiliations:** Office of the Dean and Head of Campus Te Tari Manutaki, Department of Psychological Medicine, University of Otago, PO Box 7343, Wellington South, 6242 New Zealand; Department of Psychological Medicine, University of Otago, PO Box 7343, Wellington South, 6242 New Zealand; Department of Primary Health Care and General Practice, University of Otago, PO Box 7343, Wellington South, 6242 New Zealand; Biostatistical Group, Dean’s Department, University of Otago, PO Box 7343, Wellington South, 6242 New Zealand; Department of Psychiatry, University of Ottawa, 1145 Carling Avenue, Ottawa, ON K1Z 7K4 Canada; Department of General Practice and Primary Health Care, University of Auckland, Private bag 92019, Auckland Mail Centre, Auckland, 1142 New Zealand; Registered psychotherapist in private practice, Wellington, New Zealand; Formerly at Compass Health Primary Health Organization, Wellington, New Zealand

**Keywords:** brief intervention, guided self-help, Māori mental health, mental health, mild to moderate psychological distress, primary health care

## Abstract

**Background:**

Although mild to moderate mental health problems are common and often debilitating, treatment options in primary care settings in New Zealand are often severely limited for patients with these conditions. Previously, we developed an ultra-brief intervention (UBI) to address mild to moderate psychological concerns, designed to be delivered by primary care clinicians. Recent feasibility testing, including an adaptation for Māori individuals (the indigenous people of New Zealand), showed that the brief intervention was feasible and acceptable to both clinicians and their patients. This protocol describes a large pragmatic randomized controlled trial of our UBI in primary care settings across the greater Wellington region, compared with practice as usual.

**Methods/Design:**

We are using a two-arm cluster randomized controlled trial, with primary care practices randomized to exclusively deliver either the UBI or practice as usual to all their recruited participants. The structured, guided self-help UBI is delivered in three brief general practitioner (GP) appointments over a five week period. Participants are invited into the study based on partner primary health organization access criteria (youth, people with low income, or people with Māori or Pacific Island heritage). Improvements in mental health from baseline to post-treatment will be compared between the intervention and control groups using a mixed-models application of analysis of covariance. Data analysis will be on an intention-to-treat basis, to increase the real-world relevance of UBI and to meet the study's objective of releasing UBI to primary care clinicians nationwide.

**Discussion:**

The UBI is a first-line intervention tool for GPs that models the stepped care approach advocated in New Zealand, against a background of limited access to treatments for often-overlooked patient groups. It is proposed to be accessible to clinicians and patients alike, with the potential to be relevant to primary care clinicians across New Zealand.

**Trial registration:**

Australian New Zealand Clinical Trials Registry ACTRN12613000041752.

## Background

The recently released Global Burden of Disease Study (GBD 2010) highlighted mental health and substance use as leading causes of disability and a worldwide health priority [1]. Mental and substance use disorders now represent 7.4 % of disability-adjusted life years [2] and 6 of the 20 leading causes of disability are attributable to mental health issues [3]. In line with international findings, just under 40 % of the New Zealand population had met criteria for a diagnosable mental disorder during their life, and roughly a fifth had experienced a mental disorder in the previous year [4].

In New Zealand, as in other OECD countries, mental health problems are very common in primary care and general practice. It is estimated in New Zealand that 50–70 % of mental health concerns are managed exclusively at the primary care level [5]. Primary mental health care is thus appropriately concerned with the promotion of mental health, and the prevention, assessment, early intervention, treatment and ongoing management of mental health concerns [5].

Primary care services in New Zealand are expected to respond to more of the mental health needs of the population, as secondary services become more targeted towards severe and enduring mental illness. However, treatment options for mental health problems at the primary care level are limited [5]. Between 26.5 % and 29.8 % (men and women, respectively), of primary care patients in New Zealand are thought to have mild to moderate mental health concerns by their GPs [6]. People with these problems can experience significant impairment in functioning and suffering [7, 8], indicative that treatment is required [9]. Individuals with ‘minor’ or mild to moderate depressive symptoms can have similar levels of functional impairment and place similar demands on mental and physical health services as those with more recognized diagnoses, such as major depression [10]. Some individuals with mild to moderate mental health issues will go on to develop severe depression [11, 12].

Unsurprisingly, there is a large unmet need for treatment options for common mental health concerns in primary care services in New Zealand [5, 13]. Although GPs identified that 90 % of patients who received an explicit diagnosis received treatment for mental health needs, as few as 22 % of people with mild to moderate mental health symptoms received any form of formal help [14].

It is important that primary care provides services to those who are disadvantaged in terms of access to mental health care. This includes those at socioeconomic disadvantage and many of those from Māori (the indigenous people of New Zealand) and Pacific Island populations [4]. Rates of common mental disorders in general practice settings are higher for Māori than non-Māori, although Māori are less likely than non-Māori to have been identified by their GP as having mild to moderate symptoms (15.3 % versus 23.2 %) [15].

Primary care practitioners need a range of interventions to call on, and evidence is emerging for ‘ultra-brief’ and self-help interventions, such as bibliotherapy, for mild to moderate syndromes [16–18]. It is critical that interventions are developed that are suitable for delivery by clinicians in primary care settings without the need for referral to other professionals, such as counsellors. This aids accessibility in that many people may feel more comfortable discussing concerns with a trusted GP than with a ‘counsellor’. Furthermore, referral to external services may not be possible, owing to waiting lists, criteria for service entry, or due to a lack of funds. It is also important that such interventions can be accommodated within the usual time frames of general practice consultations. A standard individual consultation in many OECD countries would last between 10 and 15 minutes, though consultation for any mental health problem might be spread over three or four visits.

In keeping with this unmet need for treatment, we developed and feasibility-tested an ultra-brief intervention (UBI) for mild to moderate mental health problems in New Zealand primary care [19]. The UBI uses a guided self-help format, involving three brief, structured face-to-face sessions with a GP, supported by self-help booklets across four topics: stress management, relationships, harmful behaviors, and bodily stress. These topics were selected because 36 % of general practice attendees report anxiety, depression or substance use, or a combination of these issues [6]. In addition, symptoms of bodily stress (previously referred to as medically unexplained symptoms) are common presentations in primary care settings [20, 21], and are being considered as a new diagnosis (ICD-11-PHC) in the forthcoming International Classification of Diseases [22]. This was reflected in GP feedback that indicated a need for resources on bodily stress. The face-to-face intervention and booklets were adapted for Māori providers and patients, and were found to be relevant and acceptable to both clinicians and their Māori patients [23, 24].

The UBI fits with a stepped care approach to mental health care in New Zealand, in which interventions range from self-help to professional help-seeking, depending on symptom severity and response to treatment. The UBI requires minimal training, no specialist counselling knowledge and incorporates techniques already familiar to most primary care clinicians. It requires only one hour of face-to-face time with the patient in total. Furthermore, it can feasibly be delivered in primary care without referral to another professional, therefore improving accessibility and acceptability of treatment for patients. In a recent feasibility test of the UBI, high levels of acceptability among primary care clinicians and patients were found, and the UBI yielded significant clinical improvements at 3 month follow-up, for both non-Māori [19] and Māori [23].

Despite evidence for the individual elements within the UBI, there has been no randomized controlled trial of the effectiveness of such a therapy delivered routinely in primary care. Thus, the aim of this trial is to investigate the clinical effectiveness of UBI for individuals who present with mild to moderate mental health needs in primary care settings compared with practice as usual, in a randomized controlled trial in a real-world study design. Specifically, we have the following objectives:

1. To compare mental health state (as measured by K10 scores) at 26 weeks between the UBI and practice-as-usual study arms. This is the primary outcome measure.
2. To compare levels of distress (depression and anxiety) and functioning (work, social and relationship) at 8 and 12 weeks between the UBI and practice-as-usual study arms. These are the secondary outcome measures.

## Methods/Design

We are using a pragmatic two-arm single-blinded, cluster randomized controlled trial of the UBI for mild to moderate mental health concerns compared with practice as usual, in a primary care setting. General practitioners have been randomized by practice to exclusively deliver either the UBI or practice as usual to all their recruited participants. The GPs are treated as the clusters in the study design (while there will be clustering by GP practice, the GPs are being treated as the unit of analysis, as practitioner attribute is anticipated to be a higher source of variability in outcomes.)

### Setting

The study is being conducted in general practices in the greater Wellington region, New Zealand.

### Participants

This is a real-world trial supported in the framework of existing treatment services. Patients aged between 18 and 65 who are identified by their GPs in a routine appointment as experiencing distress and needing a mental health intervention are eligible to participate. All participants must also score 35 or less on the Kessler 10 (K10) assessment scale, which measures global psychological distress, during their initial GP consultation. These scores were selected to indicate mild to moderate levels of psychological distress. There is no lower cut off score, since initial inclusion is based on the GP identifying a clinical problem that would be managed at the GP level. Previous work in New Zealand general practice using the K10 has demonstrated that clinicians do not select patients with very low K10 scores (<20) for clinical intervention [5]. The K10 demonstrated sensitivity and specificity for anxiety and depressive disorders, where scores exceeding 30 indicate a ‘very high’ risk of having a mental disorder [25]. This study follows previous study protocols [19, 23], where scores falling between 30 and 35 on the K10 were indicative of mild to moderate levels of psychological distress rather than a diagnosis of major psychiatric disorder, and were therefore included. Individuals receiving treatment with anti-depressant or other psychiatric medications are eligible to participate in the study.

Exclusion criteria for patients are non-fluency in English (as the intervention is an English-language based ‘talking therapy’), significant levels of cognitive impairment, as determined by the GP, and recent or acute suicidal ideation (i.e., within the previous 2 weeks). Chronic low level suicidality being managed by the GP does not exclude an individual from participating, except for those individuals who have high current levels of distress, indicated by K10 scores exceeding 35 at baseline. General practitioners are informed of patients who have high scores or suicidality at screening, or for whom referral to appropriate secondary mental health services is indicated, and these patients would not be eligible to participate further in the study. Participants are still eligible to participate if their K10 scores increase above 35 during the study, and if, in the active intervention group, they have also completed two out of three sessions of the UBI.

Inclusion criteria are based on the access criteria of a local partner primary health organization to psychological therapies. The criteria for access are specifically targeted at youth (defined as 18 to 24 years old), and, for individuals aged 25 years or older, patients with low income, or Māori or Pacific Island heritage. In line with the focus on access for youth to mental health interventions, two youth-specific services were also included in the study. The GP practices were eligible for inclusion in the study if they were members of the local partner primary health organization: no further inclusion or exclusion criteria were applied at the practice level.

### Recruitment methodology and randomization

Initial recruitment of GP practices was with the support of the partner primary health organization. The GPs were identified using primary health organization and practice lists. All of the GP practices contracted under the partner primary health organization were contacted (*N* = 52) and invited to participate in the study, and an effort was made to contact all of the GPs within these practices by email, telephone or in person. A total of 21 practices consented to participate in the study. To safeguard against gross imbalances in number of GPs per study arm, a cluster randomization schedule was created, with randomization restricted by the numbers of GPs in each practice (two medium-size practices were combined in one stratum to balance one very large practice). Of the 63 GPs who originally consented to participate, 59 completed a single two-hour training session (previously described [19]) and returned a signed consent form. Practices agreeing to take part were randomly assigned to provide either the brief intervention or practice as usual to eligible patients attending their surgeries. To ensure approximately equal numbers of GPs per study arm, randomization of practices was conducted within five blocks, according to the number of participating GPs: one, two, three, four, or more than four GPs. An additional two practices dedicated to youth health that are not part of the partner primary health organization were included and randomized into each arm of the study (i.e., these two practices formed their own stratum). Practices assigned to the practice-as-usual study arm will receive training in the intervention at the end of the study.

### Recruitment procedures

During routine clinical practice, GPs identify people with common mental health problems, who might fulfil study criteria. The diagnosis of common mental health problems is made by the GP. These patients are screened for eligibility (using the K10), and referred to the study. A research assistant then recruits participants by conducting the informed consent procedures and collects pre-treatment (baseline) data. Measures are then collected by mail or email after treatment (8, 12, and 26 weeks). Participants receive compensation (NZ $30 US $21 vouchers, a draw for an iPad) following completion of the final questionnaire, to recompense for time and effort in participating in the study.

### Intervention

The UBI is a structured and guided self-management program, and can be delivered by a GP after a single two-hour training session. Participants who have consented and completed the intake data collection (K10 and baseline measurements) will receive the GP-led intervention in three short, structured face-to-face sessions (one 30 minute and two 15 minute sessions) over a five to six week period. The UBI is a self-help approach based on structured problem solving and cognitive behavior therapy and is supported by self-help booklets on relationships, bodily stress, breaking habits and stress management. Booklets are provided to the patient after the first session, to be used in the following session. The intervention resources have been adapted to better meet the needs of Māori participants, and include Māori imagery, Te Reo (language), wairua (spirituality), whanau (family) and whakatauki (proverbs), the face-to-face sessions can include whakawhanaungatanga (the process of forming connections), and offer karakia (prayer) at the start. Measures will be collected by the research assistant before (baseline) and after treatment (8, 12, and 26 weeks). Participants can revert to practice as usual following the completion of the brief intervention. Participants can commence mental health medications at any time, or withdraw from the study and change to another intervention pathway during UBI, if deemed clinically necessary by their GP or other healthcare providers. Participants are considered to have completed the intervention if they attended at least two out of the three sessions with their GP. Increasing K10 scores during the study are not a reason for withdrawal for either intervention or control group.

### Practice as usual

Participants in the practice-as-usual study arm will receive GP support delivered according to their practice (and available existing services) as usual. Practice as usual typically consists of supportive counselling in a 15 minute face-to-face consultation, the provision of psychotropic medication, referral to psychological or other counselling options, or referral to relevant community services. All measures will be collected according to the same timescale and methodology as the intervention arm. The UBI is excluded as a component of practice-as-usual arm.

### Outcome measures

The primary outcome measure is the Kessler Psychological Distress Scale (K10) [26, 27] score at 26 weeks (adjusted for score at baseline: see analysis). The K10 is widely used as a clinical outcome measure in primary care and general practice [5]. Higher K10 scores indicate a greater likelihood of meeting criteria for a mental disorder diagnosis [27], meaning reductions in K10 scores post-treatment will represent real change in mental health status.

Secondary outcome measures are:

1. Hospital Anxiety and Depression Scale (HADS) [28]. This measures severity of depressive and anxious symptoms in outpatient hospital settings [28]. Reductions in HADS score will indicate reduced anxiety and depression. Given the nature of ‘sub-threshold’ common mental disorder, the HADS score will use the combined anxiety and depression subscales. Patients in the study are likely to have lower scores than those that might be used for categorical case definition; hence, scores have been used as continuous variables.
2. Comparison of K10 scores by treatment group at 8 weeks and 12 weeks, adjusted for baseline scores (to capture short- and medium-term effectiveness).
3. Work and Social Adjustment Scale [29]: a measure of work, social and relationship functioning) at baseline, 8, 12, and 26 weeks.
4. Sociodemographic data will include the NZDep2006 [30], a New Zealand index of individual socioeconomic deprivation.

All clinicians will be asked to complete measures of satisfaction with the intervention at the end of the study, and a randomly selected group of 20 patients will be asked to complete measures of satisfaction with the intervention at 6 weeks post-intervention. These data will be used to inform further operational development of the tool, should the result of the trial affirm its value to clinical practice.

### Statistical methods

#### Sample size and power analysis

Sample size was calculated using a simulation approach, based on standard deviations from a sample of similarly eligible patients from the UBI development study (standard deviation of post-treatment scores = 7.5; unpublished data). For 80 % power to detect a difference in K10 improvement scores of 6 points in the UBI arm, compared with 2 points in the control arm, would require 15 GPs per arm, each recruiting eight completing patients on average (*n* = 240 total with complete data). Adjusting for loss to follow-up of 20 % gives a recruitment target of ten patients per GP. The simulation settings roughly correspond to an intraclass correlation of 0.15 for considering the clustering of patient scores due to GPs: this corresponds to the intraclass correlation from the unpublished data from the development study. A power analysis for the secondary HADS scale outcome indicated that the study would have 80 % power to detect a difference of 3.2 points between groups (based on a standard deviation for the total score of approximately 6 for a general practice sample [31], with the same sampling design, and assuming a similar intraclass correlation for the HADS scale as used for the K10 measure (empirical data not available)).

#### Analysis

The statistician performing data analysis will be blinded to the intervention or control status of participants (both practices and patients). For the primary outcome, K10 scores at 26 weeks will be compared between the intervention and control groups using a mixed linear models application of analysis of covariance (comparing post-intervention scores between groups, adjusting for intake score as a covariate, and treating GP clusters as random effects). Data analysis will be conducted on an intention-to-treat basis. Missing data for the 26-week follow-ups will be handled through the mixed linear models approach to the data, which allows for participants with missing data for the final follow-up to be included in analyses (this in effect estimates a final outcome value conditional on the observed data at other follow-up times.) This missing data method is valid under the assumption that the missing observations are missing at random, conditional on the observed data [32, 33]. The null hypothesis for this test is that the K10 scores at 26 weeks (adjusted for baseline score) are not different in the intervention and control groups.

To account for stratification by type of practice (youth-health focused practices, in contrast to unrestricted GP practices) we will conduct a sensitivity analysis looking at data just from the unrestricted GP practices (i.e. excluding the two practices in the youth-health stratum).

For the secondary analysis, differences in mean scores on the K10 outcome will also be reported at 8 and 12 weeks (using the same methods as before, within the mixed linear models framework). Analysis of the HADS combined score will utilize the same methods as for the K10 outcome.

The findings of this study will be reported in line with the CONSORT statement, as adapted for cluster randomized trials [34].

### Confidentiality and data management

Consenting participants are explained their rights and provision for data confidentiality. Paper and digital copies of the data are secured in locked storage on the premises of the University of Otago, Wellington. The questionnaire data will be de-identified and entered into a spreadsheet for subsequent analysis.

### Ethics issues

Adverse events are not anticipated in this trial, and arrangements have been made to feedback clinical information to primary care GPs if deemed necessary (e.g.*,* high K10 scores, or concerning self-reported statements about a participant’s safety, in the course of data collection).

### Ethical approval

Ethical approval was received from the Health and Disability Ethics Committee of the Ministry of Health (Northern B Health and Disability ethics committee 12/NTB/2).

## Discussion

The study, due to report its findings in 2016, tests the clinical effectiveness of a UBI for common mental health concerns in primary care. The objective of this trial is to determine whether a brief psychological treatment (the UBI) delivered by practitioners in primary care settings in New Zealand is effective in reducing the disability and distress associated with mild to moderate mental health problems, by improving functioning, symptoms and quality of life, compared with practice as usual.

One potential strength of UBI for clinical practice is that an empirically derived brief structured psychological intervention for primary care could reduce the distress and disability associated with mild syndromes. A randomized controlled trial will also strengthen the evidence base for the appropriate clinical management of mild to moderate mental health conditions in primary care. The UBI might be more cost-effective in improving clinical outcomes than current practice, as it can be delivered in primary care settings without referral to another professional, by clinicians without specialist training, and using inexpensive materials. The UBI might also reduce the financial burden of seeking expensive, external, and over-subscribed services outside of primary care settings. It empowers individual practitioners to address psychological concerns in primary care settings without the reliance on mental health medications, which might not be otherwise indicated. The UBI is also a culturally sensitive intervention, tailored to the indigenous Māori peoples of New Zealand [23].

If the effectiveness of the intervention is supported by this trial, it will make a significant difference to clinical practice and patient outcomes in New Zealand. The UBI will contribute to workforce development, by up-skilling GPs to deliver the intervention. No specialist psychotherapy knowledge is needed to deliver the UBI, minimal training is required for GPs, and, furthermore, the UBI incorporates techniques already familiar to many primary care clinicians.

The UBI further benefits the community, as it is a first-line intervention tool for addressing commonly occurring mental health concerns in primary care settings. The UBI is consistent with the contemporary primary care stepped care approach that tailors interventions to symptom severity and response to treatment. It might also improve access to treatment for patients who would not otherwise seek external support. Feasibility studies have identified that the UBI leads to substantive short term improvement in mental health [19]. Furthermore, the findings of the intervention are known to be acceptable to, and were suggestive of clinical efficacy for, Māori patients. Patients with Māori heritage reported a high level of satisfaction after completing the sessions, and with the adapted imagery and Te Reo Māori included in the booklets [23]. This work has the potential to contribute to more equitable outcomes for Māori with mild to moderate mental health needs, and to empower GPs to work in a bi-culturally safe and skillful way.

A potential limitation is that the study is only open to patients who meet the funding criteria for the partner primary health organization. In real terms, participants aged 25 or older can only participate in the study if they hold a community services card or are of Māori or Pacific heritage. Applications for additional funding are underway, to increase access into the study for the general population. A result of the restricted eligibility criteria is a necessary extension of the study timeframe, which might impact the rate of recruitment and attrition of GPs participating in the trial. Despite the challenges inherent in any full-scale trial, our vision is that the UBI will form part of the standard skillset for all primary care clinicians in New Zealand.

## Trial status

Recruitment to the Stress in Primary Care (UBI) study commenced in May 2013. Participants are expected to complete all follow-up measures in late 2015.

## Abbreviations

GP: general practitioner
HADS: Hospital Anxiety and Depression Scale
K10: Kessler Psychological Distress Scale
NZDep2006: New Zealand index of individual socioeconomic deprivation
UBI: ultra-brief intervention
